# Finding Semirigid Domains in Biomolecules by Clustering Pair-Distance Variations

**DOI:** 10.1155/2014/731325

**Published:** 2014-05-15

**Authors:** Michael Kenn, Reiner Ribarics, Nevena Ilieva, Wolfgang Schreiner

**Affiliations:** ^1^Section of Biosimulation and Bioinformatics, Center for Medical Statistics, Informatics, and Intelligent Systems (CeMSIIS), Medical University of Vienna, Spitalgasse 23, 1090 Vienna, Austria; ^2^Institute for Nuclear Research and Nuclear Energy (INRNE), Bulgarian Academy of Sciences, 72, Tzarigradsko Chaussee, 1784 Sofia, Bulgaria

## Abstract

Dynamic variations in the distances between pairs of atoms are used for clustering subdomains of biomolecules. We draw on a well-known target function for clustering and first show mathematically that the assignment of atoms to clusters has to be crisp, not fuzzy, as hitherto assumed. This reduces the computational load of clustering drastically, and we demonstrate results for several biomolecules relevant in immunoinformatics. Results are evaluated regarding the number of clusters, cluster size, cluster stability, and the evolution of clusters over time. Crisp clustering lends itself as an efficient tool to locate semirigid domains in the simulation of biomolecules. Such domains seem crucial for an optimum performance of subsequent statistical analyses, aiming at detecting minute motional patterns related to antigen recognition and signal transduction.

## 1. Introduction

Molecular dynamics (MD) can be used to investigate functional elements in biomolecules [1–5]. In addition to static structures (such as crystal structures stored in the protein data bank (PDB) [6]) molecular dynamics yields information on dynamic properties [7, 8], lending themselves for evaluation of, for example, signal transduction. However, key patterns of motion related to such a functional element may be hidden among a large amount of “other” movements, reflecting no more than ordinary thermal motility of the biomolecule. Molecular dynamics itself can be carried out along relatively standardized protocols [9, 10]. However, recognizing specific patterns of motion, which are deemed crucial for a functional element, remains a tricky task, requiring sophisticated statistical methods [11], such as principal component analysis [12, 13] or normal mode analysis [14].

For all the mentioned approaches, an initial and essential step is the “fitting” of the molecular structure of each time step of an MD trajectory (henceforward called frame) to a reference structure, **x**_ref_ [15]. A given frame **x**_*i*_ is first translated to let its centre of mass coincide with that of the reference frame. Then **x**_*i*_ is rotated (around its centre of mass) to minimize square deviations between corresponding atoms of **x**_*i*_ and **x**_ref_:

(1) $$\begin{array}{c} \text{RMSD}\left(x_{i},x_{\text{ref}}\right)={\left[\frac{1}{\sum_{i}w_{i}}\overset{N}{\underset{i=1}{\sum }}w_{i}{||x_{i}-x_{\text{ref}}||}^{2}\right]}^{1/2}⟶\min. \end{array}$$

In many approaches, RMSD has been used not only for fitting but also for directly (and successfully) quantifying molecular deformations [15], including structural changes, drifts, and trends [16]. In many cases, however, even sophisticated statistical methods fail, when applied to MD-frames after fitting. The suspicion is that the process of fitting itself might cause this failure. How does this come about?

By default, the GROMACS [17] fitting procedure uses atomic masses as weights for superposition of a structure's atomic coordinates to a reference structure. Accordingly, the fitting of **x**_*i*_ “as a whole” is being optimized. In some cases, fitting the whole molecule may be inadequate and even conceal what one is searching for. For example, consider a molecule with one or more flexible loops. While the body of such a molecule behaves like a slightly deformable, rigid body, a loop may be conformationally flexible exhibiting largely uncorrelated movements with respect to the rest of the molecule. In the fitting criterion, however, atoms within the loop and those in the body may have equal weights. Since all deviations enter quadratic into (1), large movements of an even small number of loop-atoms may generate dominant contributions to the RMSD. In such a case, in order to minimize total RMSD, **x**_*i*_ is rotated predominantly to accommodate for the few atoms within the loop. As a result, the large remaining body of the molecule has to “follow its own loop,” as if the tail chases the dog [maybe this was not the primary intention of fitting]. Needless to say, due to such movements caused by fitting, that minute motile elements may become totally submerged, without any chance of being retrieved from the trajectory, not even by sophisticated statistics.

The described situation is typical and demands more elaborate fitting methods. Choosing unequal weights suggests itself as a nearby and convenient solution. The more rigid parts of the molecule should receive more weight, the flexible ones less. However, how should one know, prior to fitting, which parts are semirigid and which are flexible?

One possibility would be a two-pass procedure, in the first pass fitting to the whole molecule with uniform weights (*w*_*i*_ = 1) and evaluating the RMSF_*i*_:

(2) $$\begin{array}{c} {\text{RMSF}}_{i}=\sqrt{\langle {\left(x_{i}-x_{i,\text{ref}}\right)}^{2}\rangle }, \end{array}$$

where 〈〉 denotes the average over a trajectory and **x**_*i*,ref_ denotes a reference position of atom *i*, not changing over time. Note that RMSF_*i*_ will highly depend on the choice of the reference position, which is usually the mean coordinate of atom *i* over the whole trajectory. Then, in a second pass of fitting, weights are chosen inversely proportional to the RMSF_*i*_, as reported by [18]. Highly motile atoms receive less weight and lose their role in shaking the remaining main parts of the molecule. However, this method suffers from the fact that RMSF_*i*_ depends on the selection of **x**_*i*,ref_ in the first pass of fitting; that is, the correction procedure depends on the error it is supposed to correct.

Another possibility is the identification of semirigid domains (clusters) within the molecule, as reported by [18]. In particular, the definition of clusters may be based on distances *d*_*ij*_ = ||**x**_*i*_ − **x**_*j*_|| between pairs of atoms rather than coordinates computed in the trajectory. The standard deviation of distance variation (STDDV) between an atomic pair (*i*, *j*) is given by

(3) $$\begin{array}{c} S_{ij}=\sqrt{\frac{N}{N-1}\langle {\left(d_{ij}-\langle d_{ij}\rangle \right)}^{2}\rangle }, \end{array}$$

where *N* is the number of atoms, 〈〉 denotes the average over a trajectory, and *S*_*ij*_ is measured in nm. Evidently, pair-distances are unaffected by any kind of arbitrariness due to fitting.

Given a number of clusters (*N*_clust_), let *c*_*im*_ denote the partial class membership of atom *i* in cluster *m*. For normalization we require

(4) $$\begin{array}{c} \overset{N_{\text{clust}}}{\underset{k=1}{\sum }}c_{im}=1. \end{array}$$

The following criterion has been proposed to identify an optimum decomposition into a given number (*N*_clust_) of clusters [18]. Minimize the target function:

(5) $$\begin{array}{c} q\left(c\right)=\overset{N_{\text{clust}}}{\underset{m=1}{\sum }}\overset{N}{\underset{i=1}{\sum }}\overset{N}{\underset{j=1}{\sum }}c_{im}c_{jm}S_{ij}=\mathrm{tr}\left(c^{T}Sc\right)⟶\min \end{array}$$

under the constraints of (4). Once identified, any such cluster may be used as “primary fitting domain,” by assigning large weights to the atoms therein. With little motion within such a cluster, the motility of the remaining atoms of the molecule will appear relative to that cluster. This generally increases the chance of tracing relevant patterns of motion outside the cluster, for many statistical methods being applied.

The important difference from the known structure-analyzing tools of GROMACS, whether based on RMS deviation after fitting or RMS deviation of atom-pair distances, is that there is no need of a reference structure here. Also the clustering algorithms themselves, though with different criteria, assign conformations to a cluster for the molecule as a whole, while in our work, groups of atoms are assigned to the same cluster if their mutual distances vary little over time (a spatial clustering within the molecule).

MD trajectories for protein complexes were analyzed by clustering of averaged standard deviations of distance variation (STDDV); see below. Obtaining the most rigid cluster of atoms can be seen as the first step to facilitate the search for protein motions.

## 2. Methods

### 2.1. Construction of Complexes for Molecular Dynamics Simulation

We applied the clustering algorithm to a series of molecular systems as follows, see Table 1.

LC13 T cell receptor (TCR) in complex with major histocompatibility complex (MHC) HLA-B*44:05 and the ABCD3 peptide (EEYLQAFTY) has been successfully crystallized by Macdonald et al. [20] and its structure is accessible on http://www.pdb.org/ assigned the PDB ID* 3KPS*. However, there are no structure files of LC13 TCR in complex with HLA-B*44:02 and HLA-B*44:03. Therefore, we applied homology modelling to create these structures.

**Figure 1:**
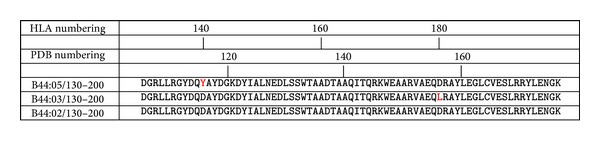
Alignment of amino acid sequences of HLA-B*44:02, HLA-B*44:03, and HLA-B*44:05 (downloaded from IMGT/HLA database [19]). HLA-B*44:05 was used as a template for homology modeling, because a three-dimensional structure of this MHC in complex with ABCD3 peptide and LC13 TCR was available. Sequence alignment was done with CLC bio's CLC sequence viewer. Note that sequence numbering from PDB (PDB numbering) and IMGT/HLA database (HLA numbering) differ.

**Figure 2:**
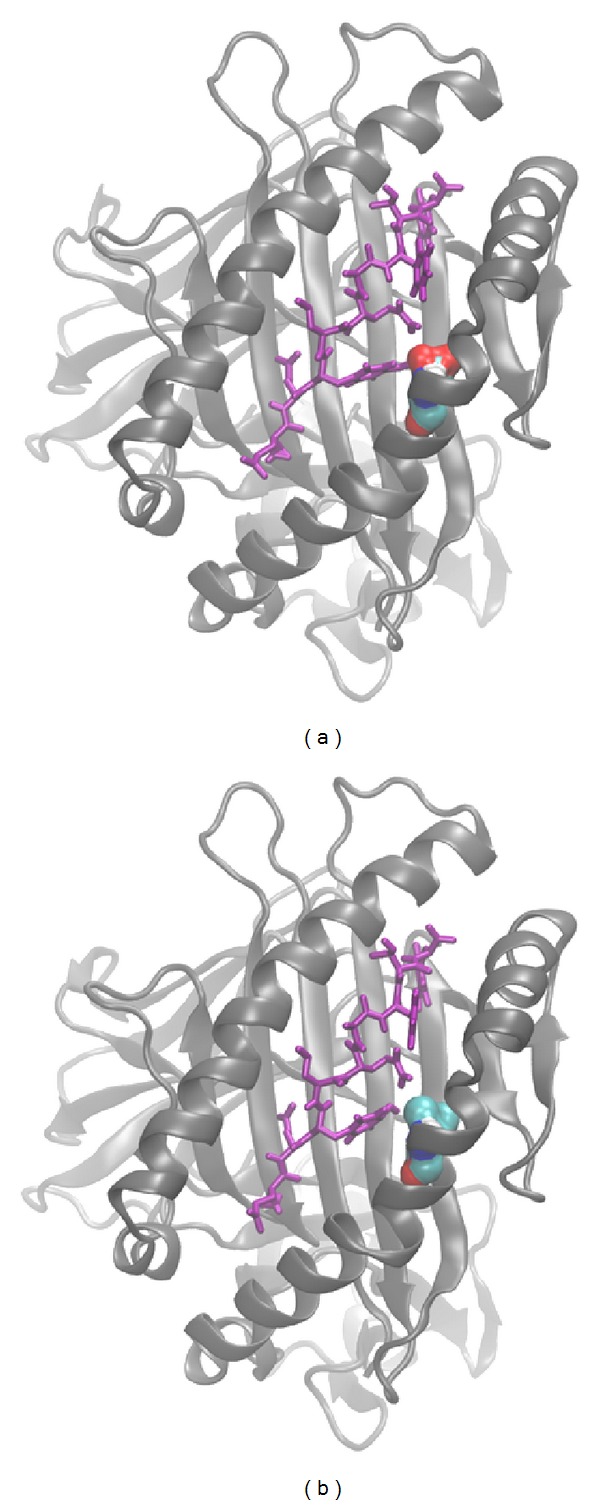
Visualisation of the D156L mutation in the MHC molecule. MHC molecules (gray) HLA-B*44:02 (left), and HLA-B*44:03 (right) together with ABCD3 peptide (violet) are shown. The amino acids comprising the D/L polymorphisms at position 156 are shown in surface representation (red*⋯*oxygen, blue*⋯*nitrogen, turquoise*⋯*carbon, white*⋯*hydrogen). Parts of the ABCD3 peptide closely interact with residue 156(D/L).

In-silico mutagenesis was carried out using Swiss PDB Viewer [21]. For generation of LC13/ABCD3/HLA-B*44:03, we used PDB structure* 3KPS* as a template and introduced mutations Y116D (numbers according to PDB numbering) and D156L to the MHC thus changing the HLA type from B*44:05 to B*44:03. For generation of LC13/ABCD3/HLA-B*44:02 we used PDB structure* 3KPS* as a template and introduced mutation Y116D to the MHC thus changing the HLA type from B*44:05 to B*44:02; see Figures 1 and 2.

### 2.2. Molecular Dynamics Simulation Protocol

The workflow of the molecular dynamics simulation of the penta-L-alanine system is closely related to that in the work of Bernhard and Noé [18]. MD simulation of penta-L-alanine was performed using GROMACS 4.0.7 [17] according to the following protocol.

First, we immersed penta-L-alanine in an explicit SPC [22] artificial water bath (cubic box) allowing for a minimum distance of 1 nm between peptide and box boundaries. Second, we minimized the solvated system using a steepest descent method. Next, we warmed up the system to 293 K during a 100 ps position restraint MD simulation. Finally, we carried out the MD production run with LINCS constraint algorithm acting on bonds with hydrogen atoms using an integration step of 2 fs and the GROMOS96 53a6 force field [23]. Coordinates were written to the trajectory every 2 ps. Coulomb interactions were computed using Particle Mesh Ewald (PME) with a maximum grid spacing of 0.12 nm and interpolation order 4. Both, Van der Waals and Coulomb interactions were computed with a cut-off at 1.4 nm. Berendsen temperature coupling to 293 K and Berendsen isotropic pressure coupling to 1 bar were used. All further parameters were set in accordance with Omasits et al. [24].

MD simulation of TCR/pMHC systems was performed using GROMACS 4.0.7 [17] according to the following protocol. First, we immersed the TCR/pMHC complex in SPC [22] artificial water bath (cubic box) allowing for a minimum distance of 2 nm between complex and box boundaries. Second, we added sodium and chloride ions to a concentration of 0.15 mol/L, and at the same time neutralizing the net charge of the system. Third, we minimized the energy of the solvated system using a steepest descent method. Next, we warmed up the system to 310 K during a 100 ps position restraints MD simulation. Finally, we carried out MD production runs with LINCS constraint algorithm acting on all bonds and using the GROMOS96 53a6 force field [23]. Hydrogen motions were removed allowing for an integration step of 5 fs. Coordinates were written to the trajectory every 50 ps. Coulomb interactions were computed using Particle Mesh Ewald (PME) with a maximum grid spacing of 0.12 nm and interpolation order 4. Both, Van der Waals and Coulomb interactions were computed with a cut-off at 1.4 nm. Velocity rescale temperature coupling to 310 K and Berendsen isotropic pressure coupling to 1 bar were used. All further parameters were set in accordance with Omasits et al. [24].

### 2.3. Optimization of Cluster Membership

Each atom *i* in a molecular dynamics simulation may be uniquely assigned to one of the *N*_clust_ clusters considered [7, 25, 26], represented by a “crisp” vector of cluster-membership; for example, *c*_*im*_ = [0,0, 0,1, 0,0] if atom *i* belongs to cluster 4 out of *N*_clust_ = 6 clusters. Alternatively, each atom *i* may be considered to belong to several clusters simultaneously, represented by fuzzy, noninteger memberships, with normalization condition see (4). Fuzzy memberships are the more general case, it seems that they might yield lower minima of the target function than crisp memberships and should therefore be preferred. Interestingly, Bernhard and Noé [18] report that fuzzy memberships, upon optimization with a gradient method, tend to end up as crisp, that is, either 0 or 1. We have scrutinized this issue and will demonstrate how this comes about. Even more, as one of the main results of this work, we will prove that the solution has to be crisp. The proof is given via mathematical arguments; see results section. This finding allows us to restrict the search space to crisp memberships, without diminishing the generality of the optimization problem posed.

#### 2.3.1. Optimization of Crisp Cluster Memberships by a Two-Stage Monte Carlo Method

Initially, the number of clusters, *N*_clust_, is chosen and the target function (5) has to be minimized. We will show that, under certain assumptions (see Section 3.1), it is sufficient to search in

(6) Ω∗={cim ∣ 1≤i≤N,1≤m≤Nclust,cim∈{0,1},  ∑m=1Nclustcim=1}.

In a less formal formulation, the objective is to assign each of the *N* atoms to one particular cluster (crisp memberships).

It may become quite tricky to attack such a problem with an analytic gradient approach since the boundary conditions are usually difficult to handle and the search domain consists of isolated points. We have therefore chosen a two-step search, in which a random process is succeeded by an exhaustive search.

Every constellation (i.e., Monte Carlo trial for improvement) which cannot be improved by a single move of one of the *N* atoms from one cluster to another is considered a result (minimum constellation). The result with the lowest *q*(**c**) is the ground state, but all other minimum constellations should also be included in the further analysis.

#### 2.3.2. Search Algorithm

*(i) Start-Up*. Initially, each of the *N* components is randomly assigned to one (of the *N*_clust_) cluster.

*(ii) Random Search*. In the first step, each of the *N* components (atoms) is moved from its current cluster to another randomly chosen cluster with probability *P*. If this mutation yields a reduction in *q*(**c**) the new constellation is preserved; otherwise, it is rejected. This process is repeated *K* times. A very rudimentary benchmarking analysis has shown that *P* = 1/*N* and *K* = *N* · *N*_clust_ are reasonable values to use.

*(iii) Exhaustive Search*. In the second step, the algorithm tries to improve *q*(**c**) by single step moves for each component separately. If there is no possible move to improve *q*(**c**), the constellation is necessarily a local minimum in our sense.

*(iv) Ground State*. Usually there is a large number of minimum constellations in the above sense. For a matrix with significant structure, the ground state will be reached after only a few trials. For ill-conditioned matrices (those without structure), a minimum constellation very close to the ground state will also be found after a few trials, although the absolute ground state might be difficult to find.

## 3. Results

### 3.1. Crisp Cluster Membership as a Necessary Consequence

We formulate and prove a lemma that crisp memberships are a necessary consequence of the topology of the multidimensional space of pair-distance standard deviations.

Due to its definition, **S** is a symmetric, nonsingular *N* × *N* adjacency matrix whose entries are the standard deviations explained earlier (3); thus, *S*_*ij*_ > 0 for *i* ≠ *j* and *S*_*ii*_ = 0. Let

(7) $$\begin{array}{c} \Omega =\left\{c_{im}\;|\;1\leq i\leq N,1\leq m\leq N_{\text{clust}},c_{im}\geq 0,\overset{N_{\text{clust}}}{\underset{m=1}{\sum }}c_{im}=1\right\}. \end{array}$$

The objective is to find $\hat{c}=\text{argmin}\;q(c)$ for **c** ∈ *Ω*.

#### 3.1.1. Lemma

If **S** is a symmetric, nonsingular *N* × *N* matrix with nonnegative entries and $\hat{c}=\text{argmin}\;q(c)$, for **c** ∈ *Ω*, then ${\hat{c}}_{im}\in \left\{0,1\right\}$.

To prove this lemma one uses Lagrange multipliers:

(8) q(c11,…,cN·Nclust,λ1,…,λN) =∑m=1Nclust∑i=1N∑j=1Ncim  cjm  Sij+∑i=1Nλi(∑m=1Nclustcim−1)⟶min⁡.

Since *q*(**c**) is a polynomial of order 2, the derivatives with respect to *c*_*im*_ and *λ*_*i*_ yield a system of *N* × (*N*_clust_ + 1) linear equations of the form:

(9) $$\begin{array}{c} \left(\begin{array}{ccc} \begin{array}{cc} S & 0\\ 0 & S \end{array} & \begin{array}{c} \cdots \\ \cdots \end{array} & \begin{array}{cc} 0 & I_{N}\\ 0 & I_{N} \end{array}\\ \begin{array}{cc} \cdots & \cdots \end{array} & \cdots & \begin{array}{cc} \cdots & \cdots \end{array}\\ \begin{array}{cc} 0 & 0\\ I_{N} & I_{N} \end{array} & \begin{array}{c} \cdots \\ \cdots \end{array} & \begin{array}{cc} S & I_{N}\\ I_{N} & 0 \end{array} \end{array}\right)\cdot \left(\begin{array}{c} \begin{array}{c} c_{1}\\ c_{2}\\ \cdots \end{array}\\ \begin{array}{c} c_{N_{clust}}\\ \lambda \end{array} \end{array}\right)=\left(\begin{array}{c} \begin{array}{c} 0\\ 0\\ \cdots \end{array}\\ \begin{array}{c} 0\\ 1 \end{array} \end{array}\right) \end{array}$$

with *I*_*N*_ being *N* × *N* identity matrix and **c**_*m*_ = (*c*_1*m*_,…, *c*_*Nm*_). The determinant of the matrix is −*N*_clust_det⁡(**S**)^*N*_clust_−1^ and therefore, under the assumption det⁡ (**S**) ≠ 0, there must be a unique solution, which is given by

(10) $$\begin{array}{c} c_{im}=\frac{1}{N_{\text{clust}}}\;\;\lambda_{i}=-\frac{2}{N_{\text{clust}}}\overset{N}{\underset{j=1}{\sum }}S_{ij}. \end{array}$$

Unfortunately, this solution yields the maximum of *q*(**c**) for **c** ∈ *Ω*. However, since *Ω* is convex and bounded, one knows that any argmin *q*(**c**) must be on *δΩ*. Therefore, there must be at least one *c*_*i*′*m*′_ = 0. Reducing the above system of linear equations by this constraint, one sees that the revolving system again has a unique solution:

(11) $$\begin{array}{c} c_{im^{\prime }}=\frac{1}{N_{\text{clust}}-1}\;\text{for}\;\;m\neq m^{\prime }\;\;c_{im}=\frac{1}{N_{\text{clust}}}\;\text{for}\;\;i\neq i^{\prime }\\ \lambda_{i}=-\frac{2}{N_{\text{clust}}}\overset{N}{\underset{j=1,j\neq i}{\sum }}S_{i,j} \end{array}$$

which again gives a maximum of *q*(**c**). The derivation of the value of *λ*_*i*_ is rather involved and not shown.

With the same argument as before, one can proceed by setting all *c*_*im*_ equal to zero with the exception of one *c*_*im*_ for each *m*. This iterative procedure shows that clustering with respect to *q*(**c**) leads to a unique assignment of each of the *N* components to one particular cluster.

### 3.2. Heuristic Evaluation of Solution Space around the Minima

**Figure 3:**
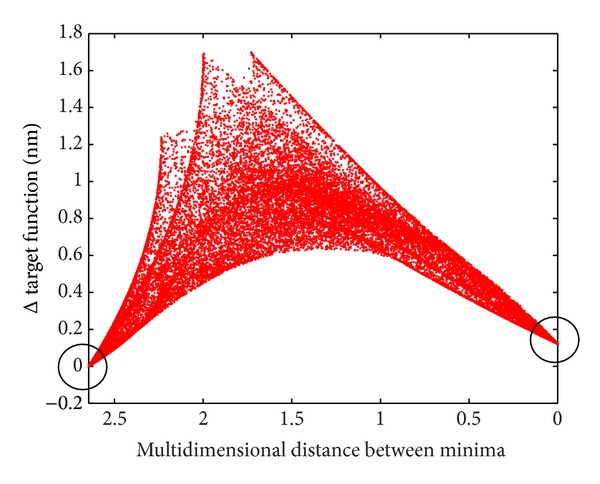
Topology of target function if a group of 7 atoms is allowed to switch between different clusters. At left and right edge of the graphics ground states are located (black circles), with corresponding values of the target function (reference minima, left somewhat lower than right). For the left state, all 7 switchable atoms are located according to cluster minimum 1, for the right state according to cluster minimum 2. In between, target function values are plotted (as red dots) for all permutations of cluster memberships for the 7 switchable atoms (3 degrees of freedom: 2 + 2 + 3 atoms). Due to the exceedingly high number of permutations, the area is densely filled with dots. Vertical axis: target function [nm] relative to minimum shown at left margin. Horizontal axis: multidimensional Euclidean distance between reference minima.

Our theoretical result, that memberships are crisp, can be illustrated very intuitively; see Figure 3. Left and right end of *x*-axis correspond to constellations with minimum target function, located at the boundary, and no other minimum is found in between.

### 3.3. Clusters of Atomic Motions in MD Trajectory

**Figure 4:**
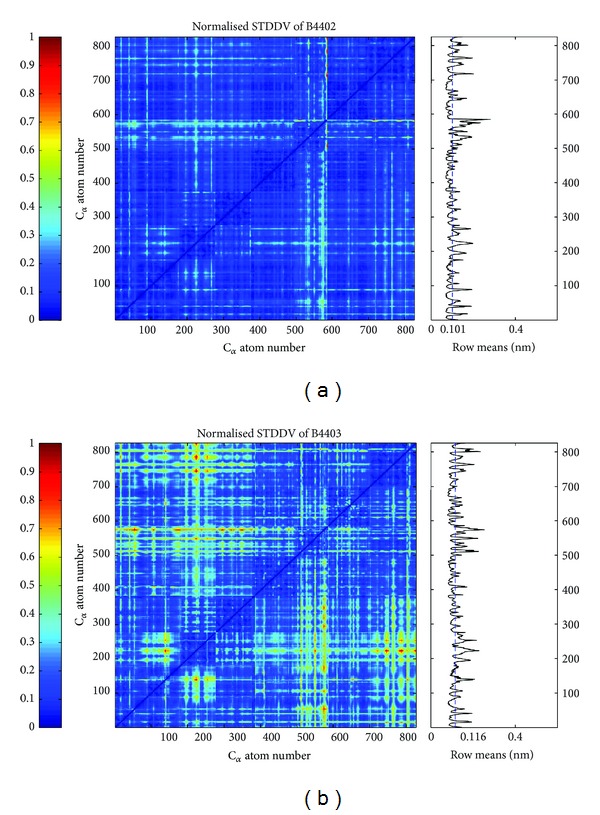
Standard deviation of pair distances (STDDV) in the second half of the trajectories of TCR/pMHC molecules B4402 (allogeneic) and B4403 (nonreactive). Values of STDDV [nm] have been normalized and are color coded (see bar on the left). Averaging over a row yields the mean distance variation against all other atoms (see subgraph on right). The dashed blue line shows the mean value of the row means, clearly indicating that the second half of the 250 ns trajectory of B4403 is more dynamic than B4402. Note that only *C*_*α*_ atoms are considered.

For above mentioned TCR/pMHC complexes B4402 and B4403, STDDV matrices were computed from the MD trajectories; see Figure 4. Only the second parts of the trajectories (corresponding to approx. 125 ns simulation time) were considered to exclude relaxation effects; see also Section 3.6.1.

**Figure 5:**
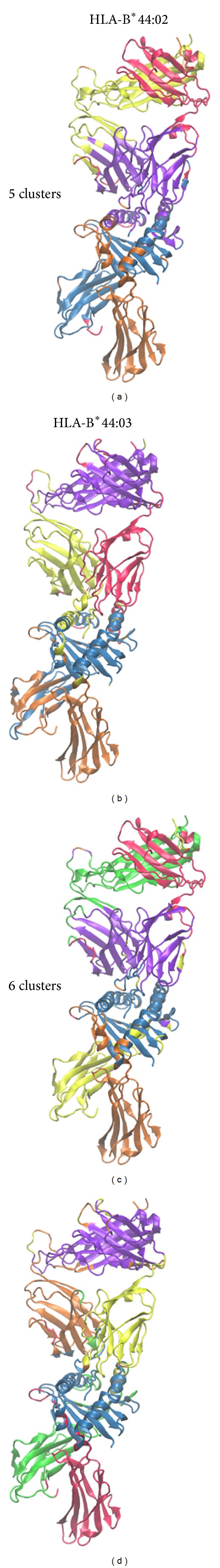
This 3D representation shows the LC13 TCR in complex with ABCD3 peptide and either HLA-B*44:02 (panels (a), (c)) or HLA-B*44:03 (panels (b), (d)). Number of clusters has been preset to five (upper panels) and six (lower panels). Clusters are rainbow-colored according to decreasing size (number of atoms in the cluster): violet (largest cluster), blue, green (relevant only for 6 clusters), yellow, orange, and red (the smallest cluster). The optimal clustering solution suggests that in these cases the most rigid clusters are the largest or the second largest ones (see also Figure 7). For panel (a) the most rigid cluster is blue and for panel (c) it is violet. For panel (b) the most rigid cluster is violet and for panel (d) it is blue. The most rigid cluster is therefore dependent on the prespecified number of clusters *N*_clust_.

STDDV matrices were clustered as described above for *N*_clust_ = 2 to 6. After computation, clusters are renumbered according to size (=number of atoms), the largest one always being labelled as cluster 1. For *N*_clust_ = 5,6 cluster memberships for B4402 and B4403 were remapped onto the protein structure and displayed in VMD [27]; see Figure 5.

Note that NO information whatsoever about secondary structural elements, such as *α*-helices and *β*-sheets, has entered the clustering procedure. Still, clusters more or less seem to retrieve some of these structural elements; see Figure 5. This could be related to extensive hydrogen bonding in *α*-helices and *β*-sheets stabilizing these secondary protein structure elements. How could bond constraints in MD simulations influence the resulting clusters? In our calculations, we just considered the protein backbone, because amino acids side chains show larger spatial fluctuations. The backbone *C*_*α*_ atoms are separated by a planar and rigid amid bond, so neighboring *C*_*α*_ atoms will experience less variation in distance.

### 3.4. More Clusters Improve Target Function

**Figure 6:**
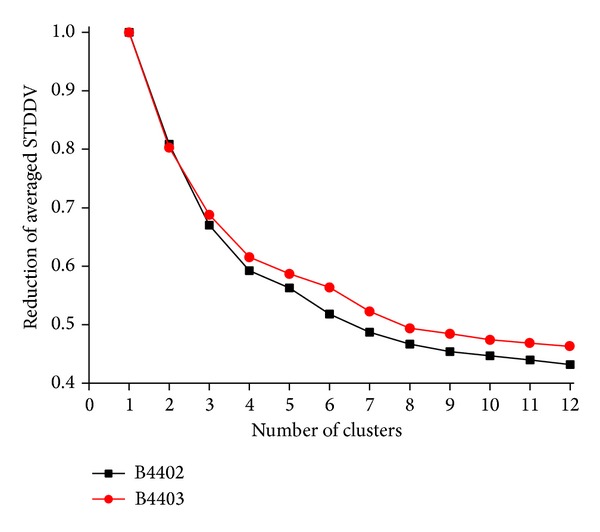
STDDV captured within clusters decreases with number of clusters. STDDV captured within clusters corresponds to the sum of areas of diagonal, coloured squares in Figure 13, right panel. Values of STDDV were normalized with respect to the total amount of STDDV without clustering (*N*_clust_ = 1) and have been multiplied by *N*_clust_ (i.e., number of squares) in order to be comparable. Note that the decline roots in the presence of mutual motile dependencies between pairs of atoms. If there were no depencencies, increasing the number of clusters would not significantly reduce STDDV, as demonstrated by the comparison with randomized dependencies shown in Figure 13.

The number of clusters has to be preselected in our approach. If we had just one cluster, the total distance variability contained in matrix** S** would be part of that cluster. Increasing the number of clusters generally reduces the fraction of variability contained within clusters, expressed as percentage of total in Figure 6.

### 3.5. Larger Clusters Turn Out to Be More Rigid

**Figure 7:**
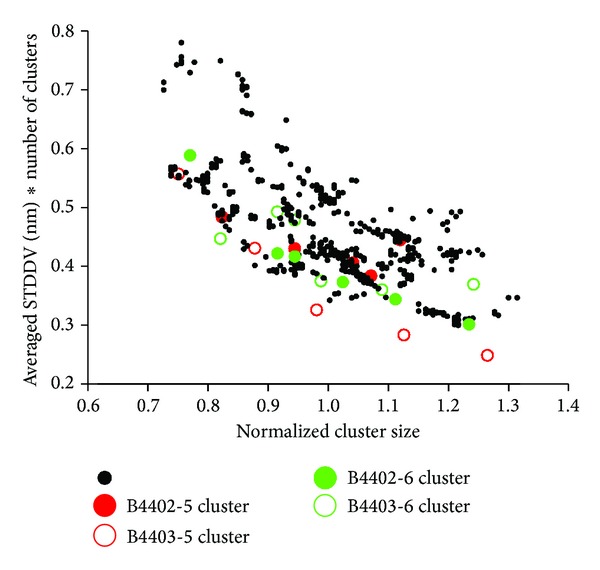
Rigidity versus size of clusters. Black data point refer to clusterings of two trajectories (B4402, B4403), each split into 50 subsections. For each subsection, clustering was performed twice, for *N*_clust_ = 5 and 6, yielding 2 · 50 · 2 = 200 runs of clustering and 50 · 2 · (5 + 6) = 1100 data points. Optimum solution for *N*_clust_ = 5 is shown in red, for *N*_clust_ = 6 in green (see Figure 5 for 3D visualization of clusters in the protein complexes). Vertical axis: STDDV within clusters was averaged and multiplied by *N*_clust_, in order to be comparable. Horizontal axis: normalized cluster size = 1 means that the number of atoms in a cluster exactly matches the average *N*/*N*_clust_.

Clusters were constructed to achieve maximum internal “rigidity,” that is, a minimum sum of pair-distance standard deviations. One might expect that large clusters, since they accommodate many atoms within larger spatial domains, should turn out to be less rigid then smaller clusters. However, the opposite is true: larger clusters turn out to be more rigid; see the declining trend of normalized STDDV with increasing cluster size in Figure 7. This demonstrates again that structures in motility are captured via clustering. If there is no structure within matrix **S**, normalized cluster sizes would result nearly equal, that is, centered around 1.

Clearly, clusters do not result identical for different subsections of a MD trajectory. In Figure 7, data are shown for two trajectories and 50 subsections each, each clustered for *N*_clust_ = 5 and 6. For details, see legend of Figure 7. 100 Monte Carlo attempts were performed for each clustering, out of which the optimum (smallest target function) was adopted. These results confirm the general trend that larger clusters are more rigid.

**Figure 8:**
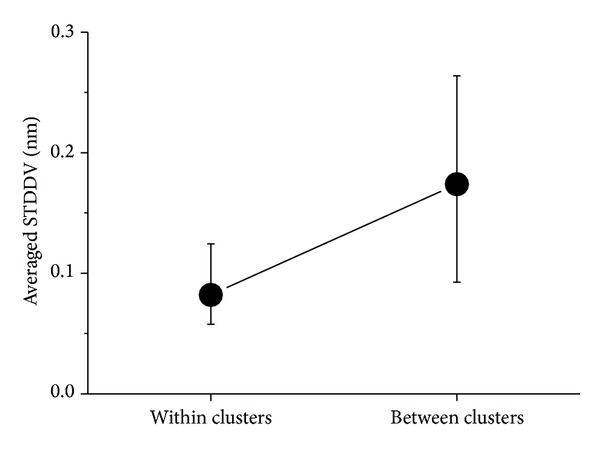
Standard deviation of pair distance variation (STDDV) within and between clusters. Symbols denote minimum, mean, and maximum.

An overview of dispersions within and between clusters is given in Figure 8, the numerical results for 6 clusters being given in Table 2.

### 3.6. Stability of Clusters

Clusters have been evaluated regarding stability, in order to check whether they lend themselves as reliable semirigid domains for fitting MD-configurations. Of note that (at least) two sources of variability of cluster memberships need to be scrutinized as follows:

- variability due to the stochastic nature of the Monte Carlo clustering method and
- variability due to different parts of an MD trajectory being clustered.

We will demonstrate that variability due to our Monte Carlo clustering method is negligible. As opposed to that, the “adequate” choice and preparation of the MD trajectory has tremendous impact and remains an issue of a never ending debate [28–32].

#### 3.6.1. Variability between Different Parts of a Single MD Trajectory

Adequate sampling of phase space is essential regarding MD-simulations [33]. Much work has been done to detect changes, drifts, and trends as markers for inadequate sampling [16, 29, 34]. Block averaging was proposed as one of the remedies [35].

In this work, the clustering presented above was based on matrices **S** computed from whole 250 ns MD trajectories. Clearly, the matrices **S**(*t*_1_, *t*_2_) for each subset (*t*_1_, *t*_2_) of a trajectory would be different, entailing different results for clustering. The question is which is the most reliable clustering for a given molecule?

To answer this question, we shall quantify the variability of clusters for subsets, relate it to the result for the whole trajectory, and derive a “stiff kernel,” that is, those atoms which do not (or very rarely) change clusters between subsets of the trajectory.

**Figure 9:**
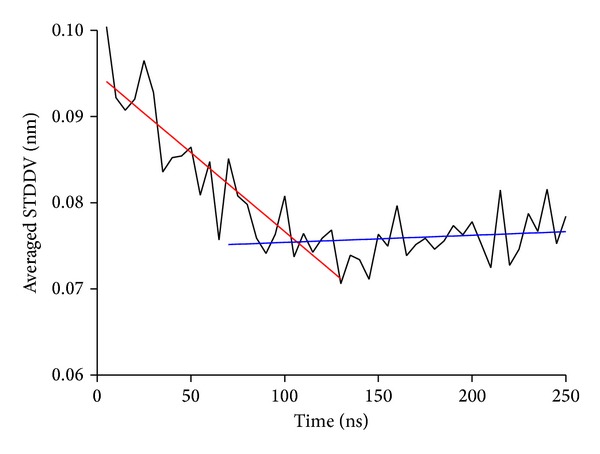
Time dependence of total variation, computed for subsets of a trajectory. Averaged STDDV (whole matrix **S**_*ij*_, example shown for B4402) computed for 50 subsets of trajectory, of 5 ns each. Straight lines have been fitted through time ranges 0–130 ns and 70–250 ns to illustrate a necessary discrimination between initial and ergodic phase of simulation.

First, we inspect the total dispersion contained within *S*_*ij*_, evaluated separately for 50 subsets of 5 ns each (i.e., 100 frames out of 5000 frames in a whole trajectory); see Figure 9. In this trajectory we observe an irregular oscillating time behavior, starting with a declining tendency. The existence of such a substantial initial phase indicates that the influence of the starting configuration does not die out until after (roughly) half of the total simulation time has passed by. To account for this fact in a heuristic way, straight lines were fitted to the first and second half of the trajectory, allowing for some overlap. This accommodates with the finding of our previous work [29] that only the second half of the trajectory can be considered an unbiased sample from phase space and should be taken for further evaluations.

#### 3.6.2. The Path along Most Stable Clusterings

**Figure 10:**
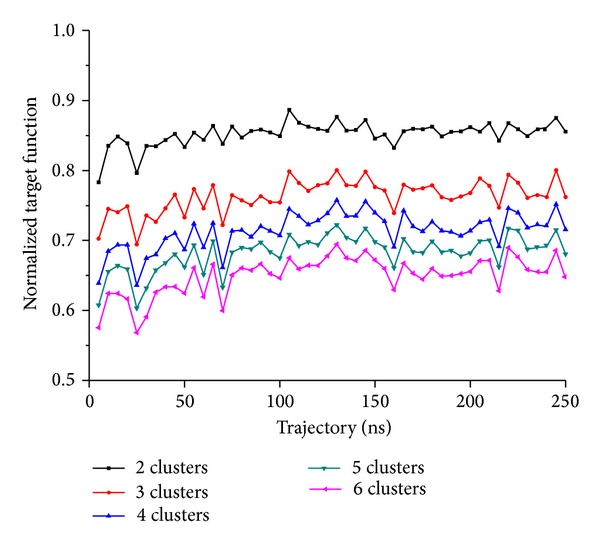
Time dependence of target function, evaluated for 50 subset-trajectories and different number of clusters. The total trajectory of 250 ns was split into 50 consecutive subset-trajectories and clustering performed for each of them, prescribing the number of clusters between 2 and 6 (see coloured legend). For each clustering, the resulting averaged STDDV is plotted against the vertical axis. Averaged STDDV have been multiplied by *N*_clust_ (as in Figure 7) to make them comparable between different numbers of clusters. Generally, more clusters entail smaller cluster size and thus reduce total motility captured in clusters. Variability of STDDV between subtrajectories illustrates the dynamical character of clustering and its dependence on the phase space sampling stage, however, with a pronounced convergence tendency of local values.

**Figure 11:**
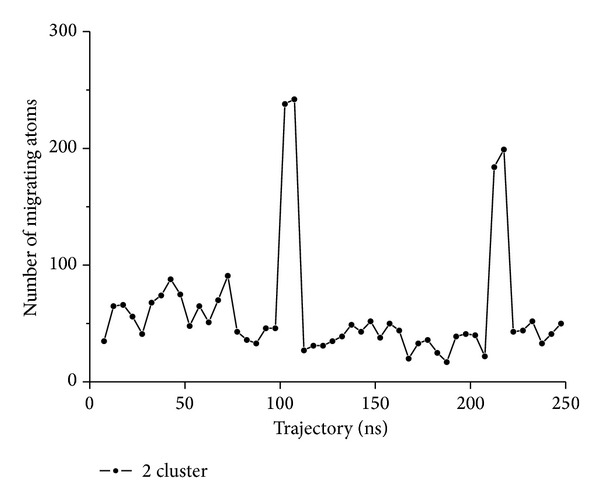
Migration of atoms between clusters. 50 subset-trajectories have been clustered (example shown for B4402, *N*_clust_ = 2). After relabelling (according to optimum permutation), the number of migrating atoms with respect to previous clustering is shown. Along this trajectory, two episodes of massive migration occur (around 100 and 220 ns, resp.). However, migrations turn out to be almost reversible; that is, most atoms finally end up in their former clusters (those of sub-trajectory 1), only about 50 (out of 826) do not.

For a preselected (number of clusters) *N*_clust_, clustering was performed for each of the 50 subset-trajectories (5 ns each), yielding an assignment for each atom to one of the clusters. Note that clusters were primarily labeled (cluster 1, 2, etc.) according to their size in that particular clustering (see Figure 10). Thus, the following situation may occur. Given a clustering of a subset-trajectory with first and second cluster about equal in size. Then, when clustering the following subset-trajectory, a few atoms from cluster 1 may end up in (i.e., “migrate” into) cluster 2, which may suffice to make cluster 2 now the largest cluster and therefore receiving the label “cluster 1” in the second clustering. This would yield a very peculiar result. The few “migrating atoms” would formally belong to the same cluster (in this example cluster 1), while the majority of atoms would switch between clusters 1 and 2. To avoid this misleading and undesired artifact, we refined the procedure as follows. In the first clustering, clusters were assigned labels (1, 2, etc.) according to decreasing size. After each subsequent clustering, we evaluated all permutations of cluster labels regarding the number of “migrating atoms” with respect to the first clustering. That permutation of labels, which yielded a minimum of migrating atoms, was finally adopted. As an example, the resulting number of migrating atoms is shown for B4402 and *N*_clust_ = 2 in Figure 11.

#### 3.6.3. Quantifying the Stability of Cluster Assignments

In Figure 11 the number of migrating atoms was considered. Now, we evaluate which atoms migrate. For quantification, we resort to the Kullback-Leibler-distance [36, 37]:

(12) $$\begin{array}{c} {\text{KLD}}_{i}=\overset{N_{\text{clust}}}{\underset{m=1}{\sum }}p_{im}\cdot \log\frac{p_{im}}{1/N_{\text{clust}}}. \end{array}$$

1/*N*_clust_ represents the assumed background probability if the assignment of atom *i* to any of the clusters were equally probable. *p*_*im*_ is the actual probability for atom *i* to belong to cluster *m*, estimated from an average over cluster memberships *c*_*im*_ obtained from clustering subsets of the trajectory:

(13) $$\begin{array}{c} p_{im}=\langle c_{im}\rangle . \end{array}$$

**Figure 12:**
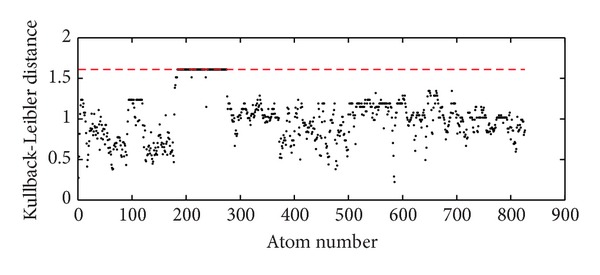
Kullback-Leibler distance as a measure for stability of cluster assignments among 50 subtrajectories. For each of the 50 subtrajectories, a separate clustering was performed. Comparing these clusterings, atoms are seen to move between the clusters several times. We consider the Kullback-Leibler distance (KLD) to estimate how far the distribution of the individual atoms among clusters deviates from a uniform distribution. Maximum possible values KLD = log_10_(50), shown as red dashed line, correspond to atoms which never changed clusters. This can be observed, for example, for atoms with indices between 200 and 300. The lower the KLD, the more often the atoms move from one cluster to another. Data are shown for B4402, *N*_clust_ = 5.

Large values of KLD_*i*_ indicate that atom *i* stays predominantly in the same cluster throughout the trajectory. On the contrary, values of KLD_*i*_ close to zero indicate a random distribution of an atom between all clusters.  Figure 12 shows KLD_*i*_ for B4402 and *N*_clust_ = 5.

## 4. Discussion

### 4.1. Clustering Reflects Structure within STDDV-Matrix

Target function (5) only counts distance variability within the clusters, not between atoms belonging to different clusters. Thus, if we reorder atoms according to their cluster membership, clusters appear as squares along the main diagonal of the matrix **S**; see Figure 15. If we hypothetically assume that elements S_*ij*_ are more or less homogenously distributed across the matrix, the “area” of each cluster in the matrix will roughly correspond to the variability within that cluster. Clearly, these squares have to be of equal size to make their joint area minimum (and thus minimize the total distance variation within clusters).

**Figure 13:**
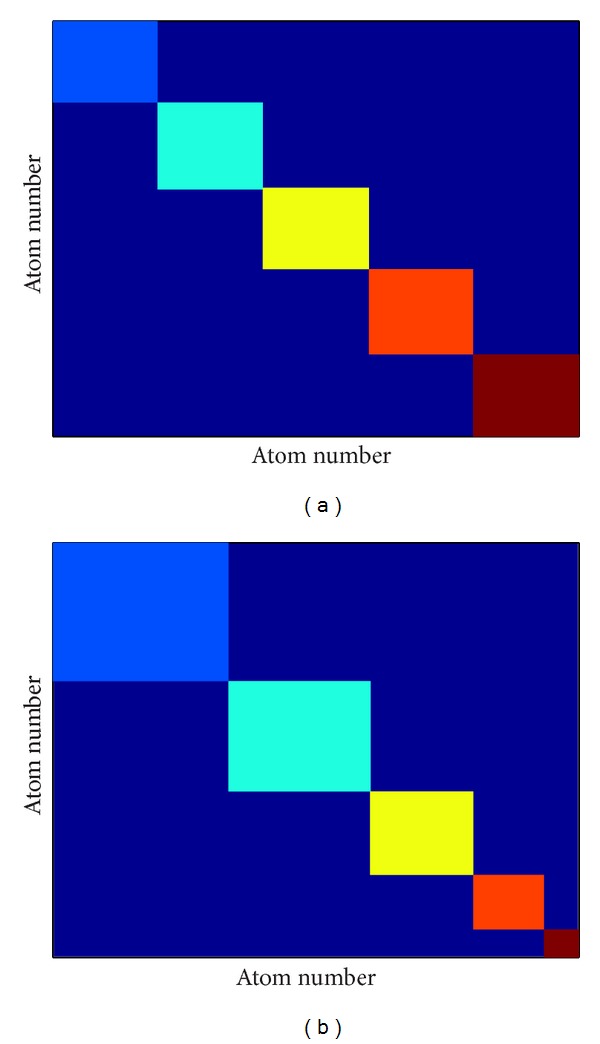
Clusters of equal and unequal sizes. If the pair-distance variations were randomly distributed, clusters of equal sizes were optimum (a). If clusters of unequal size result, this indicates that structure is present in the matrix (b). The figure is a schematic illustration for *N*_clust_ = 6.

We have verified this prognosis by randomly rearranging elements of matrix **S** and then performing the clustering procedure. Cluster sizes resulted almost equal (for illustration see left panel of Figure 13) in each of 20 trials of random rearrangement and clustering. This finding was verified for 2 ≤ *N*_clust_ ≤ 6 (data not shown).

**Figure 14:**
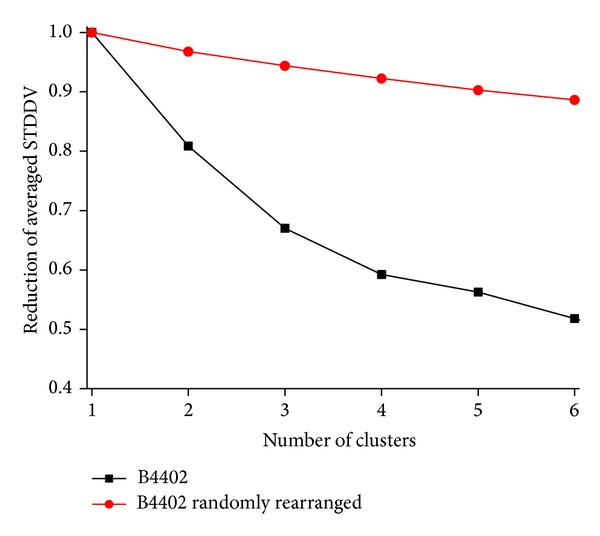
Improvement of target function with number of clusters. Comparison between data from MD trajectory B4402 and randomly rearranged matrix elements *S*_*ij*_. Black graph “B4402” relates to matrix **S** obtained from a real MD-simulation, whereas the red graph “B4402 randomly rearranged” stems from a matrix with randomly rearranged elements (STDDVs).

**Figure 15:**
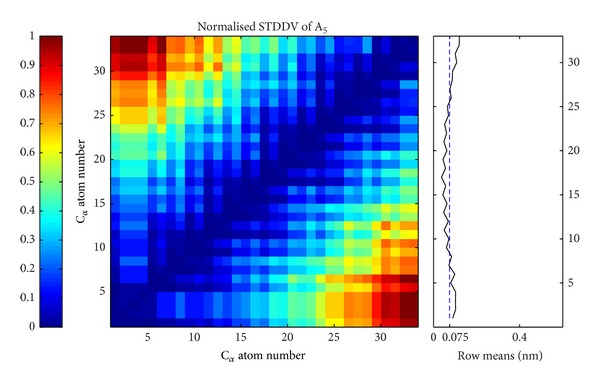
Matrix of standard deviation of distance variation (STDDV) for the A_5_ pentapeptide (33 atoms). Values of STDDV [nm] are color coded (see bar on the left). Averaging over a row yields the mean distance variation against all other atoms (see subgraph on the right).

As opposed, clusters of very different sizes resulted for matrices **S** derived from MD simulation (illustrated in right panel of Figure 13). This clearly indicates that clusters of unequal size do not result by chance but reflect distinct dependencies within **S**. The sensitivity of clustering to existing structures within **S** is also reflected in target function *q*(**S**), as can be seen by comparing matrices **S** from MD and their randomly rearranged counterparts; see Figure 14. In both cases averaged STDDV decreases (improves) with increasing number of clusters. However, in presence of real dependencies between atomic mobility, clustering achieves much more improvement.

### 4.2. Many Small Clusters Are More Rigid but Cover Less Distance Variation

One might ask why a larger cluster number is less favorable. Obviously, the extreme case of defining each single pair of atoms as a separate cluster would lead to minimum STDDV within clusters but would represent a trivial and useless solution. Note that STTDV within clusters decreases with increasing *N*_clust_, which makes clusters more homogeneous and is a desired effect. However, at the same time the overall amount of variability caught within clusters also decreases, which is an undesired effect, since larger portions of the molecule are disregarded. These trends are reflected by the total area of coloured squares along the diagonal in Figure 13. The smaller the squares (with increasing *N*_clust_), the smaller their total area, even if there are more squares (the area decreases quadratic with the side lengths of squares).

This tendency has been quantitatively demonstrated for real MD-data in Figure 6. On top of that, Figure 14 shows that the same trend also holds for unstructured matrices, as mentioned above. However, results also show that matrices without structure (randomly rearranged elements) allow for very little reduction in the STDDV covered within clusters, as compared to structured matrices and that this difference further increases with *N*_clust_.

### 4.3. Clustering Is Stable and Self-Contained

Powerful statistics on MD-trajectories (which is, e.g., able to detect small motions related to signaling) needs careful fitting of configuration frames as a prerequisite. Fitting to a domain which should be as rigid and large as possible is one of the options.

Hence, finding large clusters is desirable. However, large clusters in general might prove unstable. Therefore, we have carefully investigated this issue for the target function proposed by Bernhard and Noé [18] in conjunction with our clustering method.

It turned out that larger clusters are even more stable (see Figure 7), which is a strong indicator of stability and self-containment of our results.

### 4.4. Ergodicity

Atomic motions in a large molecule constitute a highly dimensional phase space, and MD-simulations can in most cases explore only part of the total phase space. At least, one can never be sure about the exact fraction of phase space actually visited in a specific simulation run. As a consequence we use only the second half of our MD trajectory for final clustering (see Figure 5), in accordance with our previous work [29].

### 4.5. Computational Resources

Clustering takes very little iteration steps for A_5_, due to monotonous, continuous relationships between elements of** S**; see the appendix. Note that the rapidity of clustering in this case is not only a primary consequence of the small number of atoms but a matter of simplicity in structure.

Randomized matrices** S** take significantly more iterations for clustering, since many minima are almost equal regarding the target function, rendering solutions ambiguous. As opposed to this, clustering large molecules with structured internal motion, such as B4402 and B4403, yields well-defined minima after a reasonable number of trials.

### 4.6. Cluster Interpretation

There is an obvious difference in visual appearance between STDDV matrices for B4402 and B4403 as seen in Figure 4. Trajectory B4402 yields an unstructured, rather flat STDDV matrix (upper panel in Figure 4), while B4403 shows a distinctly structured STDDV matrix (lower panel in Figure 4). The relation between cluster rigidity and size is illustrated in Figure 7 and shows a clear trend: cluster rigidity increases with increasing cluster size, reflected in a decreasing STDDV within clusters. However, this does not mean that the largest cluster is always the most rigid cluster (i.e., has lowest STDDV). For *N*_clust_ = 5, we see that in B4403 the largest cluster is at the same time the most rigid one. For *N*_clust_ = 5 in B4402 the second largest cluster is the most rigid one. For *N*_clust_ = 6 the situation is inverted: in B4402 the largest cluster is the most rigid one. In B4403 the second largest cluster is most rigid.

All in all, the most rigid cluster was always found among the two largest clusters. The mappings of the clustering results for *N*_clust_ = 5 and *N*_clust_ = 6 for B4402 and B4403 have been displayed in Figure 5.

### 4.7. Conclusion and Prospects

A main result of the paper is the finding that the target function proposed by others [18] has only crisp solutions; that is, each atom belongs to one single cluster only (and not to several clusters in a fuzzy sense). This finding allows for a much more efficient search for optimum clustering.

Based on this new finding, the process of clustering was evaluated regarding various aspects to provide concomitant information for possible application by other investigators. Applicability was demonstrated for two trajectories of 250 ns each for large biomolecular complexes whose dynamics is of key importance for the understanding of immune reactions.

Further improvements can be expected from a more detailed investigation of the Kullback-Leibler distance [36, 37]. In this work it has only been reported as a means for assessing the quality of clustering by some given method. In future work, the Kullback-Leibler-distance may enter the clustering procedure itself and render clusters even more stable between subtrajectories and over time.

## Figures and Tables

**Table 1:** Molecular complexes simulated.

Molecular system	Simulation length
Penta-L-alanine (A_5_)	1000 ns
LC13 TCR/ABCD3/HLA-B*44:02 (B4402)	250 ns
LC13 TCR/ABCD3/HLA-B*44:03 (B4403)	250 ns

**Table 2:** Dispersion of pair-distances within and between clusters.

	1	2	3	4	5	6
1	**0.058**	0.115	0.093	0.145	0.178	0.167
2	0.115	**0.068**	0.146	0.193	0.219	0.129
3	0.093	0.146	**0.079**	0.123	0.175	0.212
4	0.145	0.193	0.123	**0.075**	0.210	0.264
5	0.178	0.219	0.175	0.210	**0.090**	0.241
6	0.167	0.129	0.212	0.264	0.241	**0.124**

The full trajectory of protein complex B4402 was clustered into 6 clusters. Numbers in main diagonal give averaged STDDVs [nm] within clusters, corresponding to left markers (symbols: mean, minimum, maximum) in Figure 8. Off-diagonal values relate to STDDV between clusters, corresponding to right marker in Figure 8.

## Appendices

### A. Pair-Distance Variations in a Small Molecule

For comparison, we applied our clustering method also to the A_5_ penta-L-alanine peptide already analyzed by Bernhard and Noé [18]. They choose penta-L-alanine to evaluate their clustering algorithm on MD simulations for reasons of simplicity of this molecule. A_5_ has four amide bonds, each comprising four atoms (CONH). The delocalization of the nitrogen's free electron between conjugated carbonyl and amine groups poses planarity and rigidity onto this structure.

Mu et al. [38] showed that A_5_ does not remain in an alpha-helical conformation, but rather exhibits repeated folding and unfolding events. As expected for such a molecule, our clustering of the averaged STDDV matrix indicates that atoms close together show little fluctuations of their mutual distances, such as the atoms in the middle of the pentapeptide; they are, so to speak, in the centre of the storm. Conversely, atoms near the edges show large distance fluctuations with respect to all other atoms.

Clustering such a homogeneous matrix **S** does not reveal structural elements within the molecule, but rather splits it into equal parts, according to the number of clusters preselected. Note, however, that this behavior is a property of the equally distributed matrix values S_*ij*_ and not a general rule.

### B. Software Availability

The software used for clustering into crisp domains of preselected number is currently being implemented in Java and will be made available for free download from http://www.meduniwien.ac.at/msi/biosim/index.php?lang=en&seite=en_forLehreIt_pairDistanceClustering.
